# Hinged Adaptive Fiber-Rubber Composites Driven by Shape Memory Alloys—Development and Simulation

**DOI:** 10.3390/ma15113830

**Published:** 2022-05-27

**Authors:** Felix Lohse, Achyuth Ram Annadata, Eric Häntzsche, Thomas Gereke, Wolfgang Trümper, Chokri Cherif

**Affiliations:** Institute for Textile Machinery and High Performance Material Technology, Technical University Dresden, 01062 Dresden, Germany; achyuth_ram.annadata@tu-dresden.de (A.R.A.); eric.haentzsche@tu-dresden.de (E.H.); thomas.gereke@tu-dresden.de (T.G.); wolfgang.truemper@tu-dresden.de (W.T.); chokri.cherif@tu-dresden.de (C.C.)

**Keywords:** shape memory alloy, fiber-rubber composite, simulation, hinge

## Abstract

Adaptive structures based on fiber-rubber composites with integrated Shape Memory Alloys are promising candidates for active deformation tasks in the fields of soft robotics and human-machine interactions. Solid-body hinges improve the deformation behavior of such composite structures. Textile technology enables the user to develop reinforcement fabrics with tailored properties suited for hinged actuation mechanisms. In this work, flat knitting technology is used to create biaxially reinforced, multilayer knitted fabrics with hinge areas and integrated Shape Memory Alloy wires. The hinge areas are achieved by dividing the structures into sections and varying the configuration and number of reinforcement fibers from section to section. The fabrics are then infused with silicone, producing a fiber-rubber composite specimen. An existing simulation model is enhanced to account for the hinges present within the specimen. The active deformation behavior of the resulting structures is then tested experimentally, showing large deformations of the hinged specimens. Finally, the simulation results are compared to the experimental results, showing deformations deviating from the experiments due to the developmental stage of the specimens. Future work will benefit from the findings by improving the deformation behavior of the specimens and enabling further development for first applications.

## 1. Introduction

In recent works, Shape Memory Alloys (SMA) have proven to be highly suited for the creation of adaptive structures [1,2,3,4,5,6]. Their thermomechanically induced shape change properties enable their use as actuators, thereby the reducing size, complexity and cost of actuation-based applications by replacing conventional actuators such as electrical motors, hydraulics and pneumatics [7,8,9]. Due to the low required construction space of SMA actuators, they are well-suited for integration in fiber-reinforced polymer (FRP) composites, thus enabling the creation of functionally integrated adaptive structures with properties tailored to specific applications [10]. Due to the high stiffnesses of typical FRP based on thermoset resins, the achievable SMA-driven bending movements are comparatively small [11,12]. In order to increase the range of movements and maximum deformations, recent work has focused on rubber matrices, enabling high reversible strains during bending deformation [3,4,5].

In order to further improve the deformation behavior of such soft adaptive structures, solid-body hinges can be introduced by varying the bending stiffness across the specimen [13,14], thus concentrating the SMA-induced deformation in smaller areas. In past work by the authors [6], wire-shaped SMA were integrated into tailored reinforcement textiles by means of flat knitting technology and processed into interactive fiber-rubber composite (IFRC) specimens capable of large deformations. Additionally, a simulation model was developed, to describe the deformation behavior of the IFRC.

The presented work uses existing knitting patterns of biaxially reinforced multilayered knitted fabrics, extended towards sectioned patterns with varying numbers of reinforcement fibers to create high stiffness (HSS) and low stiffness sections (LSS) that act as solid-body hinges. Additionally, the SMA integration is extended towards sectioned patterns, enabling structures with two separately activatable hinges. The active deformation behavior of the specimens was evaluated in deformation tests, where the bending deformation was induced by thermal activation of the integrated SMA actuator. An existing mesoscopic simulation model within the framework of the finite element method (FEM) is extended with regard to the hinge sections and is calibrated and validated with experimental data.

### 1.1. State of the Art

#### 1.1.1. Shape Memory Alloys

SMA are capable of memorizing their geometrical shape after undergoing deformation. When subjected to temperature changes after an initial deformation, they tend to return to their initial shape. At high temperatures, the crystalline configuration present within the SMA is referred to as austenite. The crystalline configuration present at low temperatures is referred to as martensite. The phase transformation from austenite to martensite is a diffusion-less solid-phase transformation leading to twinned martensite with zero stress and de-twinned martensite under significant stress influence. The austenite phase is stable at high temperatures and exhibits a cubic crystalline structure whereas martensite is stable at low temperatures with tetragonal or monoclinic crystalline structures [15]. When heated, the SMA transforms from martensite to austenite. The start and finish temperatures of austenite and martensite are represented as A_s_, A_f_, M_s_, and M_f_, respectively.

The change in the crystallographic structure during solid-phase transformation exhibits mechanical behavioral changes resulting in shape memory effect (SME) and pseudo-elasticity (or superelasticity). The functional properties of the SMA material can be controlled in a wide range by means of appropriate thermal/thermomechanical processing methods as well as changing the alloy composition. The shape memory effects are further categorized into two characteristics [7]:

- One Way Shape Memory Effect (OWSME)
- Two Way Shape Memory Effect (TWSME)

For a better understanding of shape memory characteristics, it can be assumed that the SMA is existing in twinned martensite (i.e., mechanically unstrained state) at room temperature. Mechanically loading this SMA causes detwinning, thus, deforming the SMA pseudoplastically. Subsequent heating above the alloy-specific austenite forming end temperature A_f_ results in austenitic (parent-memorized) phase formation, reverting the initial pseudoplastic deformation and changing its shape back to that of the unstrained state. When the SMA is cooled back to below the martensite-forming end temperature M_f_, SMA reverts to twinned martensite state without further shape change. This effect is called OWSME. The temperature-induced OWSME exhibits large strains and—when the free deformation is hindered—large stresses, enabling SMAs to generate large deformations and transduce large forces for actuation tasks.

If after cooling, the SMA reverts back to the detwinned martensite state instead of the twinned martensite phase, this effect is called TWSME. During this two-way effect, the phase transformation can be reverted to austenite when heat is supplied under constant loading. However, to obtain TWSME, the SMA material must undergo a cyclic thermomechanical treatment denoted as “training” to remember its austenitic and detwinned martensitic states. The TWSME produces less recovery strain than OWSME and the strain tends to deteriorate quickly when subjected to high temperatures, rendering TWSME less feasible for actuation tasks [7].

Since superelasticity or pseudoelasticity is less relevant in this work, it is only mentioned briefly. Here, the SMA is utilized in the Austenite state above A_f_. Upon mechanical loading, the SMA undergoes a stress-induced solid-phase transformation towards Martensite, allowing large-degree deformations that are completely recovered after the load is discarded. Thus, recoverable strains of up to 10% can be utilized. Additionally, the superelastic strain exhibits a hysteresis, facilitating large energy dissipation capabilities of superelastic SMA. For more details on shape memory effects, refer to e.g., [7,15,16].

#### 1.1.2. Hinged Actuation Mechanisms

**Figure 1:**
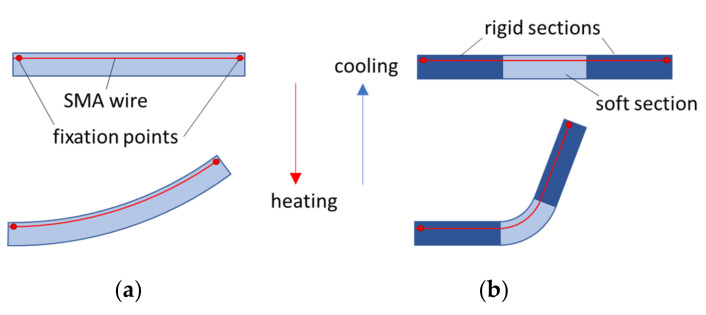
Deformation principle of (**a**) a homogenously soft bending beam and (**b**) a hinged bending beam with rigid sections and a soft middle section.

The basic concept of active deformation of a beam sample driven by contractile SMA wires studied in this work is based on bending as the primary mode of deformation. As depicted in Figure 1a, the SMA wire is integrated into the beam close to its surface (outside of the neutral axis) and fixed to the beam on both ends. In between, the SMA can move freely.

Upon heating by applying an electric current, the SMA wire transforms to austenite and contracts, thereby deforming the beam (cf. Figure 1a). Upon cooling, the wire converts back to martensite, allowing the structural stiffness of the beam to deform both the SMA and the beam back to their original, unbent shape.

As already shown in [6], the geometrical relationships of this mechanism yield three main parameters that determine the achievable deformations: beam thickness, beam length and maximum SMA contraction. Since the SMA contraction is limited by the material, thickness and length of the beam remain as parameters. Increasing the achievable deformations requires either longer or thinner specimens, which conflicts with application-related requirements such as limited installation space and high transferable forces. The design scope of simple bending beams for technical applications is therefore limited.

Consequently, introducing hinges into bending structures by splitting the deforming beam into HSS and LSS can significantly increase the achievable deformations without changing length or thickness (Figure 1b). The LSS allow the full-length SMA contraction to act on a much shorter section of the beam. This is possible due to the SMA’s free movability between its fixation points. Viewed the other way around, the length of the SMA wire and the length of the deforming section are decoupled, which resembles the approach of Lee et al. [3]. In [3], the SMA wire was extended beyond the plate length, increasing the usable actuator stroke.

Wang et al. [14,17] have shown that hinged bending structures can perform larger deformations compared to bending structures without hinges with otherwise the same geometric parameters. This is achieved by concentrating the SMA-induced bending on smaller sections of the overall structure. In the work by Wang et al., the rigid sections were generated by integrating plastic components with high stiffness, resulting in close to no deformation in the respective HSS upon SMA activation. However, the specimen developed in this work exhibit overall lower bending stiffness and thus tend to bend both in their HSSs and LSSs, although to different extents. It can be assumed that the SMA contraction is distributed between the LSS and HSS, with the ratio of distribution depending on the stiffness ratio of the sections. Thus, the higher the difference in stiffness, the higher the deformation of the LSS. For tailoring structures to practical applications, a precise dimensioning of the stiffness of LSS and HSS is therefore required.

#### 1.1.3. Knitted Fiber-Reinforced Rubbers

Unlike thermoset FRP, which provide both high stiffness and strength, IFRC specimens employ a highly elastic rubber matrix. In combination with stiff reinforcing fibers, this results in highly anisotropic mechanical properties that allow large reversible bending deformations while maintaining high membrane stiffness. IFRC structures are therefore suitable for applications under uniaxial or planar loads such as tires, pressure hoses or conveyor belts [18].

In this work, multilayer flat knitting technology [19,20] is used to manufacture biaxially reinforced fabrics with hinge sections and integrated SMA actuator wires. The highly flexible flat knitting technology is capable of tailoring the properties of reinforcement textiles to the requirements of the hinged bending structures while simultaneously integrating the SMA wires in the intended position. Thin PTFE tubes are used to coat the SMA wires against direct contact with the matrix during composite forming. The fabrics can then be molded into IFRC specimens using a vacuum-assisted resin infusion (VARI) process. This reduces the number of necessary process steps while keeping the effort required for machine configuration and programming relatively low.

#### 1.1.4. Simulation Model

In order to support both dimensioning and provide insight in the complex material interactions of IFRC structures, using a simulation model is highly beneficial. As several authors have shown [21,22,23], analytical descriptions of SMA-driven actuator structures are difficult to solve, due to the complex material behavior and component interactions. Anisotropic fiber reinforcements and solid-body hinges add further complexity.

Therefore, a mesoscopic FEM modeling approach for IFRC with integrated SMA wires was developed in ANSYS^®^ as part of a previous work by the authors [6]. In mesoscopic modeling, the reinforcement fiber yarns are described as discreet geometry integrated within the surrounding matrix. The computational effort is higher than with macroscopic modeling, where the composite properties are captured by a material model without taking the properties of the individual components into account. However, the level of detail is higher and allows for more concise investigations of the composite behavior and the interactions of its components. As shown in [6], the modeling approach overestimates the bending stiffness of the fiber-rubber composite without SMA (FRC) and therefore requires calibration with experimental data. The existing model is briefly explained below. Please refer to the original work for further details.

- **Geometry and mesh**: The model consists of a silicone block with cavities for the reinforcement fibers and SMA wire. The reinforcement fibers in the weft direction are modeled as cylinders with elliptical cross-sections and the SMA wires are modeled as a cylinder with circular cross-sections. The thin PTFE tube (cf. Section 1.1.3) surrounding the SMA wires within the specimens is neglected in the model, since stiffness is low and has no significant influence on the bending stiffness. Instead, the cylindrical cavity in the silicone is assigned the diameter of the PTFE tubes, in order to replicate the contact interactions of the wires with the tube. The reinforcement fibers in the warp direction are neglected in the geometry modeling, assuming that their influence on the bending stiffness is captured by calibration with experimental bending test data. All components are meshed with hexagonal 3D elements. The meshes of the fibers share the nodes coinciding with the silicone at their respective interfaces, assuming an ideal interface bonding. In order to reduce modeling effort as well as improve convergence behavior, symmetry is utilized. For this, it is assumed that the force transduced by the SMA wires is distributed evenly across the reinforcement fibers within the specimen. Thus, a specimen with four SMA wires and 24 reinforcement fibers is reduced to one SMA wire and 6 reinforcement fibers.
- **Material modeling**: The fiber material is modeled using an orthotropic elastic material model, with stiffness parameters determined by calibration with four-point bending test data of the composite material. The rubber material is modeled with a hyperelastic material model by Yeoh et al. [24], which was fitted to experimental data from tensile tests of rubber specimens. The SMA material is modeled with a thermomechanical material model based on the work of Auricchio and Souza [25,26]. The material parameters were obtained from thermomechanical test data of the SMA material. Tensile tests at room temperature and above A_f_, DSC measurements and isobaric contraction measurements were conducted for the characterization. The model requires pre-stretching by a virtual force in a preliminary load step in order to exhibit the shape memory effect used for actuation.
- **Boundary conditions**: The SMA wire interacts with its surrounding cavity through a frictionless contact boundary condition, emulating the smooth surface of the PTFE tube. A bonded contact boundary condition connects the free end of the SMA to an external fixation element. Another bonded contact boundary condition connects the fixation element itself to the silicone rubber. In order to account for the symmetry within the model, the side faces of the specimen are restricted from moving in a normal direction with a translational boundary condition.
- **Loads**: With a force of 15 N in the first load step, the SMA wire is pre-stretched. After stretching, the force load is discarded and a ramped temperature load is applied to the SMA geometry, initiating the contraction caused by the shape memory effect.

## 2. Materials and Methods

### 2.1. Materials

The materials used in this study are listed in Table 1. For the SMA material, E_M_ and E_A_ refer to the elastic moduli of the martensite and austenite phases, respectively. The SMA material was pre-strained by the manufacturer by cold-drawing. Additionally, a thermal treatment for long-term stability was applied. Information on the thermal treatment is not available due to the non-disclosure of the manufacturer. Two types of glass fibers were used for the biaxially reinforced fabrics—a 410 tex yarn, further denoted as **GF-strong**, and a 2 × 136 tex twisted twin yarn, further denoted as **GF-faint**. The two yarns are denoted according to their reinforcing effect on the composite. The silicone rubber material was used as a matrix to produce the composite. For further details on the material properties and characterization refer to Lohse et al. [6].

### 2.2. Concept Development for Hinged Structures

**Figure 2:**
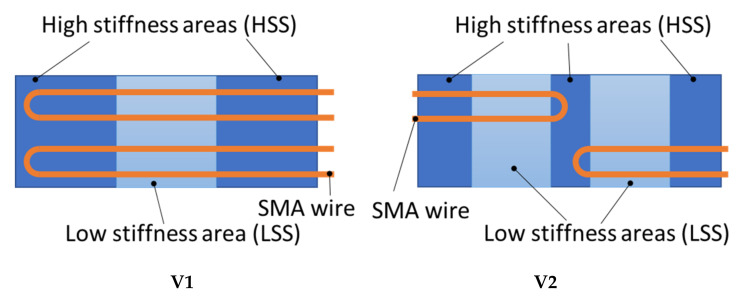
Concepts of SMA-driven hinged structures. **V1**—one hinge area (LSS) and full SMA loops; **V2**—two hinge areas (LSS) and sectioned SMA loops.

For creating hinged structures, two concepts are pursued. Both concepts are plate-shaped with the dimensions 120 × 80 mm^2^ (Figure 2).

- Concept **1** (**V1**) has a low stiffness section (LSS) in the middle and two high stiffness sections (HSS) at the edges. Integrated SMA wires are spanning the full plate length, changing direction in turn-around curves at the end. During activation, the LSS is more prone to deformation due to its lower stiffness and therefore acts like a solid-body hinge.
- Concept **2** (**V2**) has two LSS, each flanked by HSS. Furthermore, SMA wires are integrated in sections, so that two strands with turn-around curves span the left LSS, the next two strands span the right LSS, and so on. Thus, the SMA wires can be activated independently, deforming either left, right or both LSSs of the structure, thus enabling intermediate deformation states.

In order to investigate the assumed influence of both HSS and LSS being prone to bending (cf. Section 1.1.2), two variants of V1 are produced:

- V1.1 with one hinge area and two layers of reinforcement fibers in the HSS in bending direction, as described in Section 2.3.1.
- V1.2 identical to V1.1 during the knitting process but with two additional layers of reinforcement fibers in its HSS added during the manufacturing of the composite. Thus, V1.2 will have twice the amount of reinforcement fibers in its HSS compared to V1.1.

It is expected that the bending deformation of V1.2 will be more concentrated on the LSS than in V1.1, leading to higher maximum deformation.

### 2.3. Binding Development and Knitting Process

**Figure 3:**
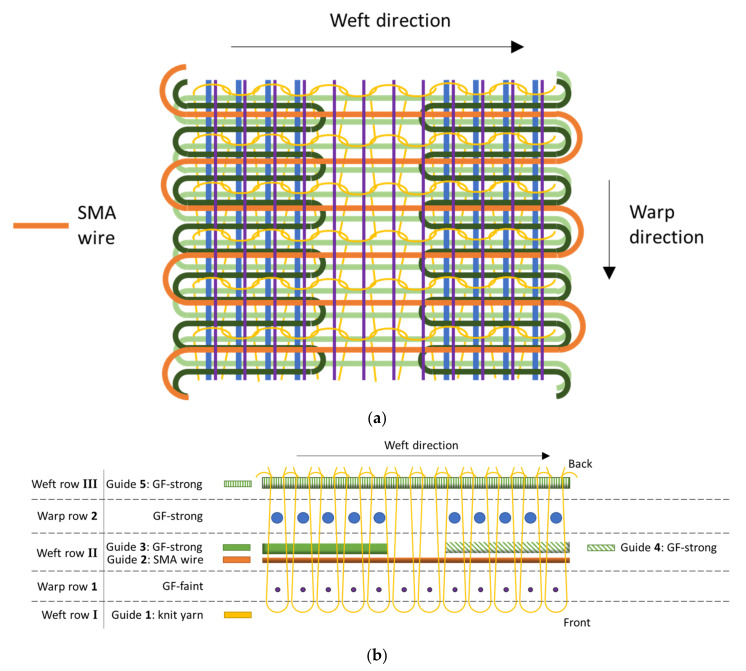
(**a**) Knitting pattern concept for V1 and (**b**) top view of the knitting unit, warp direction to the image plane. The displayed number of yarns is reduced for better visibility.

**Figure 4:**
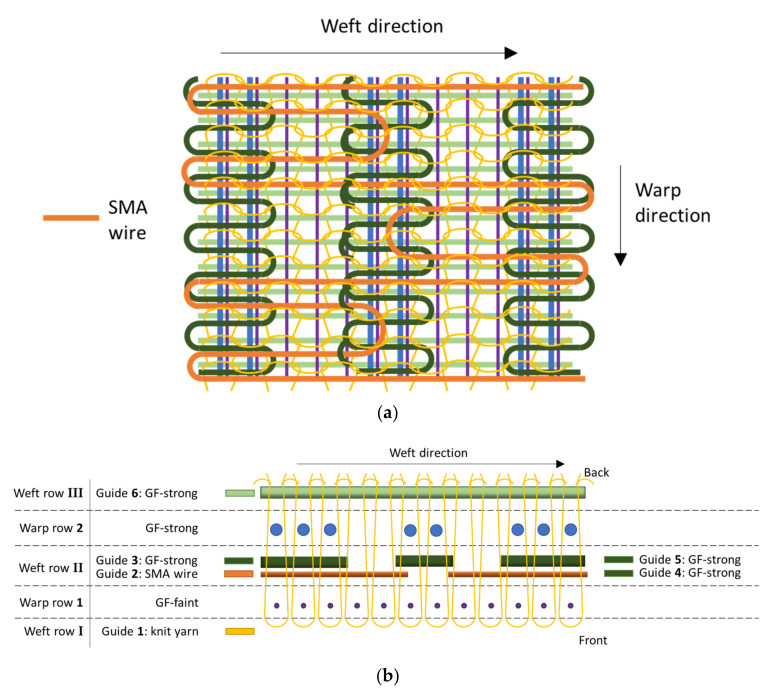
(**a**) Knitting pattern concept with customized SMA alignment for V2 and (**b**) top view of the knitting unit, warp direction to the image plane. The displayed number of yarns is reduced for better visibility.

Both concepts are realized by means of multilayer flat knitting technology. Here, one layer of the fabric holds fibers in a uniform direction without undulations, with the layers alternating between weft and warp direction. For this, a customized Aries.3^®^ flat knitting machine with a segmented fabric take-up [19] and a machine fineness of E7 is used. The SMA wires are integrated with the weft direction. The LSSs and HSSs are realized by varying the number of reinforcement fibers across the width of the knitted fabric. This is achieved by using multiple thread guides, that enable local insertion of the fibers (Figure 3a and Figure 4a). In order to minimize friction between SMA wires and rubber, thin PTFE tubes are used to cover the SMA wires. Due to their low thickness, the tubes are processed on the flat knitting machine similar to the yarn material. Thus, the SMA wires were inserted into the tubes and then processed collectively on the flat knitting machine.

#### 2.3.1. Binding Setup of V1

Two variants of V1 were produced, further denoted as V1.1 and V1.2. They share the same binding setup, with their distinct features being created during composite forming. Thus, subsequently, the binding setup for both variants is described jointly. The differences between V1.1 and V1.2 are described at the end of the section.

V1 consists of two warp rows and three weft rows. Five weft thread guides grouped in the three weft rows are used (weft row **I** in front, weft row **II** in between and weft row **III** behind the warp rows), with warp row **1** (front) holding GF-faint yarns and warp row **2** (back) holding GF-strong yarns (Figure 3b). Guide **1** in weft row **I** provides the knit yarn that binds the layers together and moves in front of warp row **1**. Guide **2** in weft row **II**, holding the SMA wire with PTFE, moves between warp rows **1** and **2** and travels over the full fabric width. In order to increase the distance between SMA wires, guide **2** only moves in every fifth stitch course, leaving four weft rows between the wires. Guide **3** in weft row **II**, which holds the GF-strong yarn, travels only part of the weft length and stops at the start of the LSS. Upon return of guide **1**, guide **3** travels back, thus alternating back and forth across the left HSS in each row. Guide **4** in weft row **II** acts similar to guide **3**, moving back and forth across the right HSS. Guide **5** in weft row **III**, holding GF- strong yarn, moves behind warp rows **1** and **2**, traveling the full length in each row.

With the LSS containing only one layer of GF-strong in the weft direction, its stiffness is expected to be significantly lower than that of the HSS with two layers of GF-strong and thus twice the fiber amount. For V1.2, blanks of GF knitted fabric identical to the layer setup of the HSS (without SMA) are draped onto the HSS, doubling the number of GF layers and further increasing the stiffness of the section.

#### 2.3.2. Binding Setup of V2

For V2, two warp rows and six weft thread guides are used, with warp rows **1** and **2** being identical to V1 (Figure 4b). Thread guide **1** in weft row **I** holds the knit yarn and moves in the front of both warp rows over the full weft length. Due to the limited number of thread guides available between the warp rows, the sectional SMA patterns are created with one thread guide by alternating positions between the left and right half of the specimen and doing side switches in between. Thus, guide **2** in weft row **II**, holding the SMA wire, is moving between warp rows **1** and **2** as follows: starting from the left edge of the fabric width across the left LSS and back, then across the whole weft length, then, starting from the right edge of the fabric width, across the right LSS and back, and again across the whole weft length and so on (cf. Figure 4a).

To separate the SMA patterns on the left and right sides, the sections of the SMA wire that pan the entire weft length are severed and manually removed. The guides **3** and **4** in weft row **II** holding 410 tex GF yarns act similar to guides **3** and **4** in V1, moving only across the left and right HSS at the edges of the structure and between warp rows **1** and **2**. Additionally, guide **5** in weft row **II**, also holding 410 tex GF yarns, moves back and forth only across the HSS in the middle. Guide **6** in weft row **III** acts equal to guide **5** in V1, holding 410 tex GF yarn and moving behind warp row **2** across the whole weft length. The fiber layer distribution of the sections is thus identical to that of V1 (cf. Section 2.3.1). The main differences are that V2 has two LSSs and the middle HSS holds parts of the SMA wire loops.

### 2.4. Composite Development and Characterization

Both V1 and V2 are manufactured into composite specimens with a vacuum-assisted resin infusion (VARI) process. Blanks of the fabric (120 × 80 mm^2^) are placed on a metal plate and wrapped into a vacuum bag with additional layers for improving resin flow and demolding. After infusing with silicone, the specimens are cured at room temperature for 48 h. The vacuum layup is listed in Table 2.

After the VARI process, the composite specimens are very thin and, as preliminary work has shown, yield insufficient deformation behavior upon activation. Therefore, an additional thin layer of silicone is poured on top of the specimen.

**Figure 5:**
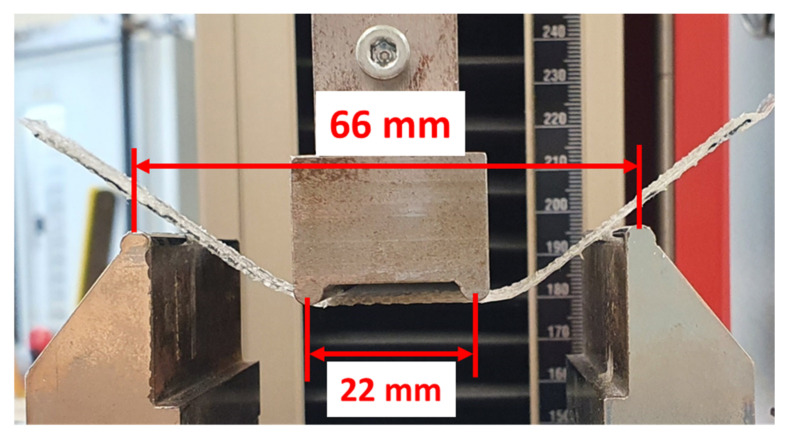
Four-point bending test for evaluating fiber-rubber specimen of V1 and V2, maximum deformation shown.

In order to evaluate the bending stiffness of the specimens V1 and V2, four-point-bending tests were carried out on a Zwick and Roell^®^ Z100 testing machine (ZwickRoell GmbH, Ulm, Germany) based on DIN EN ISO 14,125 [27]. Sheets of FRC specimens of V1 and V2 without SMA wires were used. Since the SMA is characterized separately, the data is used for calibrating the bending behavior of the FEV without SMA. The sheets have the dimensions of 15 × 130 × 0.7 mm^3^, with four GF yarns lengthwise. The specimens are tested in the weft direction, in accordance with the direction of the SMA-induced bending. The setup is displayed in Figure 5.

### 2.5. Simulation Model Enhancements

**Figure 6:**
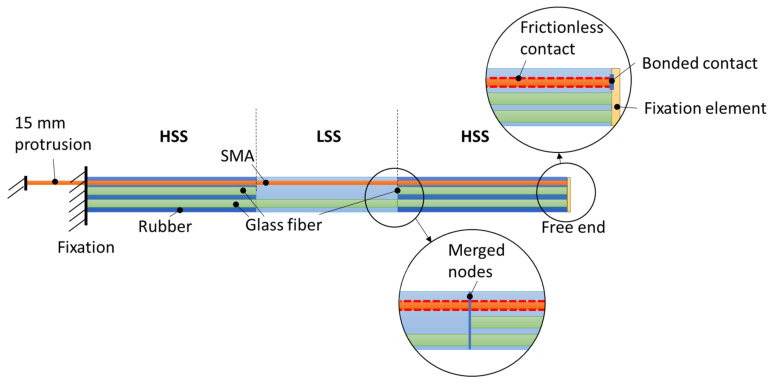
Model concept for V1 with one hinge area.

**Figure 7:**
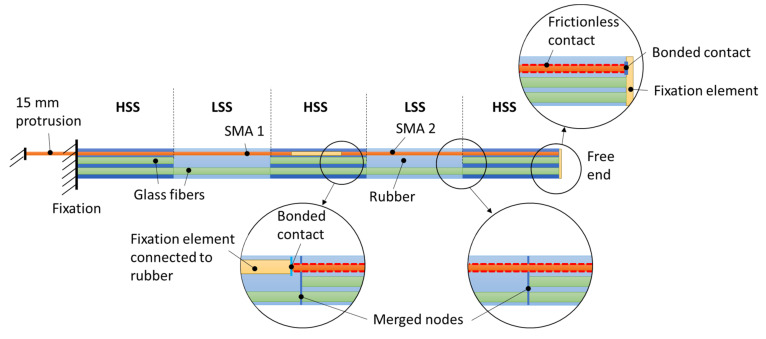
Model concept for V2 with two hinge areas and two separate SMA wires.

In order to account for the solid-body hinges present in samples V1 and V2, the existing model (see Section 1.1.4) is extended accordingly. For V1, the model is divided into three sections—two HSSs with two layers of 410 tex GF yarns and one LSS with one layer of 410 tex GF yarns (see Figure 6). For V2, five sections are generated—three HSSs and two LSSs (see Figure 7). As with the existing model, the nodes of the fiber and rubber geometry are merged at the respective interfaces, assuming ideal interface bonding. At the interfaces of LSS and HSS, the coinciding nodes are merged as well, creating a continuous structure. Furthermore, a second SMA wire is added to the V2 model, with both wires spanning half of the total specimen length.

As with the existing model, symmetry is used to reduce modeling and calculation effort, by using one SMA wire only and reducing the number of weft yarn rows to six. In addition to this symmetry assumption, the structure is cut in half, reducing the model to one-half of an SMA wire and three weft yarn rows. The resulting symmetry surface is assigned the translational boundary condition described in Section 1.1.4, restricting movements in the normal direction of the cut faces.

As with the existing model, the SMA wires need to be pre-stretched in order to exhibit the shape memory effect. This is done in two preliminary load steps, with a pre-stretch force of 7.5 N per wire (reduced by a factor of 0.5 to account for the symmetry described above). In the first step, the force is ramped up, pre-stretching the wire. In the second step, the force is ramped down, recovering the elastic deformation of the SMA and leaving the SMA in a plastically deformed (detwinned) state. For V1, the free end of the SMA wire is then connected to the structure with a bonded contact.

For V2, the pre-stretching process is more elaborate: SMA 2 is temporarily fixed with a zero-displacement boundary condition to the free end of the beam, with its free end pre-stretched towards SMA 1. SMA 1 is in turn attached to the fixed side of the beam and pre-stretched at its other end towards SMA 2. After pre-stretching, both SMA 1 and SMA 2 are connected to a central fixation element, which is in turn connected to the rubber structure. The zero-displacement boundary condition of the other end of SMA 2 is then discarded; instead, it is then connected to the fixation element at the free end of the beam with another bonded contact. The wires are then activated separately or simultaneously with temperature loads.

The bending stiffness of the FRC models without SMA needs to be calibrated with experimental data of the FRC specimen in order to ensure that the stiffness behavior of the model and the real structure agree. Therefore, the mesoscopic models of hinged FRCs without SMA wires are used to replicate four-point bending tests. In order to save computing effort, symmetry is used to model only half of the geometry. The gained results are therefore multiplied by two during evaluation. The calibration is conducted by iteratively changing the Young’s modulus in the 0° direction of the fiber material model.

### 2.6. Setup for Bending Deformation Tests by Thermal Activation of the SMA Actuators

**Figure 8:**
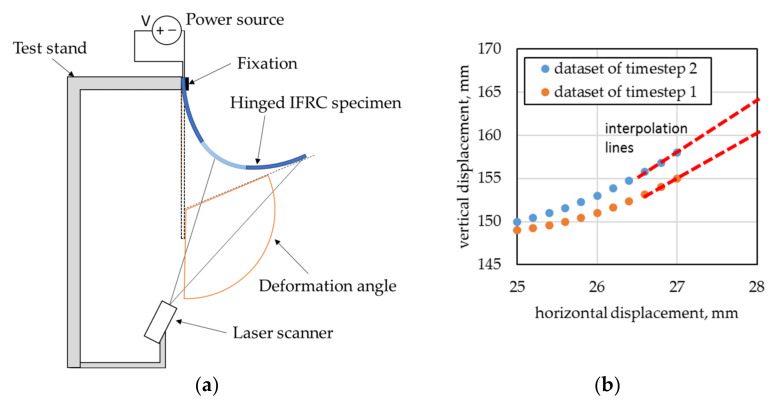
(**a**) Test stand for deformation measurements of IFRC specimen in top view, based on [6]; (**b**) principle of angle estimation by interpolating through data points of the free end of the beam.

In order to minimize the influence of gravity, specimens are clamped such that the deformation initialized due to electro-thermal activation of the integrated SMA actuator takes place parallel to the ground. The specimens are clamped on one end only. To measure the deformation, a laser scanner (LJ-V7200, Keyence GmbH, Osaka, Japan) with a resolution of 1.0 µm and a range of 152 to 248 mm is positioned to aim at the backside of the specimen. Upon thermal activation of the SMA actuator, the CCD line sensor of the scanner captures the surface of the deformed IFRC specimen (Figure 8a).

The deformation angle is obtained by first extracting the laser scanner’s data of a short section closest to the free end for each time step. A straight line is then interpolated through the measured data sets and the angle of the line relative to the starting position is calculated (Figure 8b). A programmable laboratory power supply unit (Rohde and Schwarz HMP4040) is connected with alligator clamps to the SMA wires, providing electric current for thermal activation of the actuator. For V2, the SMAs of the left- and right-hand sides are connected to separate channels of the power supply unit in order to enable consecutive activation. Different activation modes are used for the specimens:

- For V1, the voltage is set to 16 V, resulting in a current of ~2.0 A. The cycle time is set to 5 s for activation and 10 s for deactivation, with a total of five cycles.
- For V2, two modes are used:
  ○ In mode **A**, both SMA wires are activated simultaneously with 14 V, resulting in a current of 2 A due to the shorter SMA wires of V2, with 5 s activation and 10 s deactivation.
  ○ In mode **B**, the SMA wires are activated consecutively. The first SMA is activated for 20 s, the second SMA for 10 s, starting 10 s after activating the first. Then, both SMA are deactivated for 10 s.

**Figure 9:**
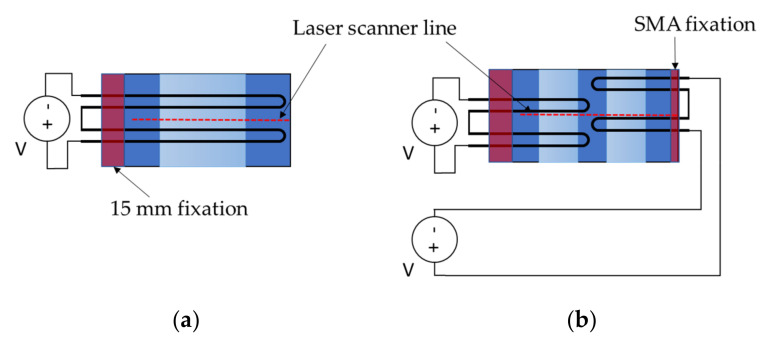
SMA clamping and power connection setup for (**a**) V1 and (**b**) V2.

Bolts, nuts and ring washers are used to clamp the SMA wires of V1 on the fixed side of the specimen. For V2, the SMAs on the fixed side are clamped in the same way as for V1. The SMAs protruding from the free end are fixed with nuts, bolts and ring washers to a low-weight clamping plate attached to the free end (Figure 9).

## 3. Results

### 3.1. Knitting Results

**Figure 10:**
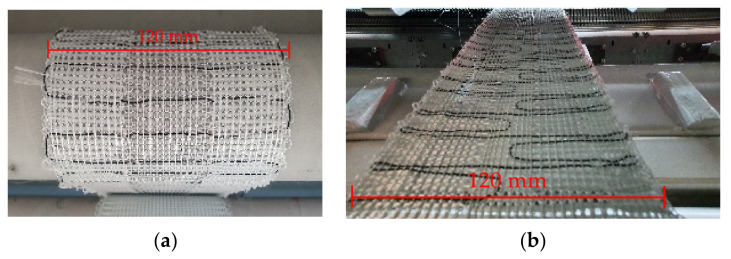
Biaxially reinforced knitted fabrics (**a**) V1 and (**b**) V2 with blank yarns instead of SMA wires.

**Figure 11:**
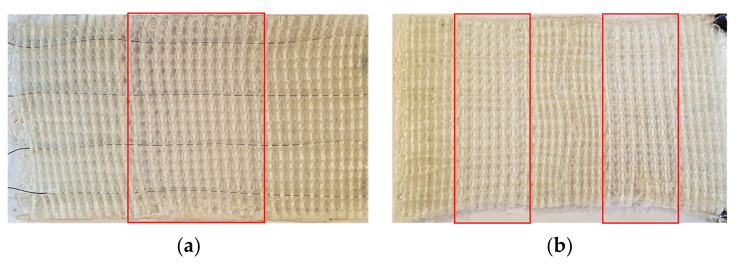
Composite specimen of (**a**) V1 and (**b**) V2 with one and two low stiffness hinge areas (LSS), respectively. LSS areas are marked in red.

The knitted fabrics for V1 and V2 are displayed in Figure 10a,b, respectively. In these figures, a fabric section with a blank yarn is used instead of SMA wires for improved visibility of the SMA alignment. Even though the SMA wires possess higher stiffness than the blank yarn, the integrated SMA wires only cause low undulations of the fibers (Figure 11).

The observable undulations are estimated as acceptable since the optimization of the mechanical properties is not the goal of the presented work. The finalized composite specimens of V1 and V2 are displayed in Figure 11a,b, respectively, with their LSS areas marked for improved visibility.

Since the fibers are compressed during the VARI process, the difference in thickness of LSS and HSS is negligible. The distinct amounts of reinforcement fibers in the respective plate sections rather than their geometry induce the major change in stiffness. The geometries of the resulting composite specimen are displayed in Table 3.

### 3.2. Results of Bending Characterization Tests

**Figure 12:**
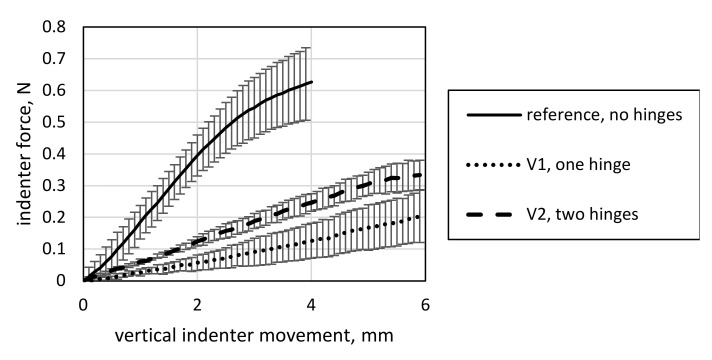
Four-point bending test data of V1 and V2, with a reference data set of a specimen with no hinges for comparison from [6].

The results of four-point bending tests for V1 and V2 are displayed in Figure 12, together with a reference specimen of a previous study [6]. The reference specimen has two layers of 410 tex GF yarns over the entire length without solid-body hinges. As the comparison with the reference data shows, bending stiffness decreases significantly by introducing hinges into the structure. Furthermore, V1 exhibits lower bending stiffness than V2, contradicting the intuitively expected result that two hinges would yield an overall lower bending stiffness. It is worth noting that the results of the four-point bending test do not directly correlate to the expected improvements in the deformation behavior. The effect can be explained by closely examining the setup. For V1, the indenters contact the specimen roughly at the beginning of the LSS. Thus, mainly the bending of the LSS is measured, which is comprised of only one layer of 410 tex GF yarns. For V2, the indenters contact the specimen at the center of its two LSSs, deforming both the LSSs and the middle HSS, holding two layers of 410 tex GF yarns.

### 3.3. Results of Bending Deformation Tests by Thermal Activation of the SMA Actuators

**Figure 13:**
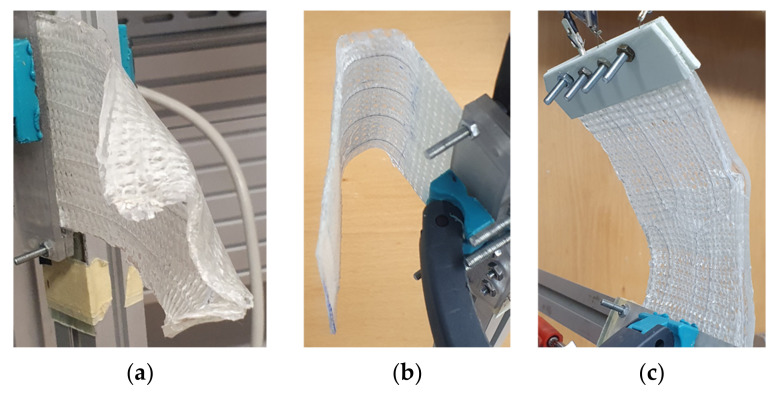
Deformed specimen (**a**) V1.1 with significant edge curling, (**b**) V1.2 with large deformation and (**c**) V2 with simultaneous activation of both SMA loops.

**Figure 14:**
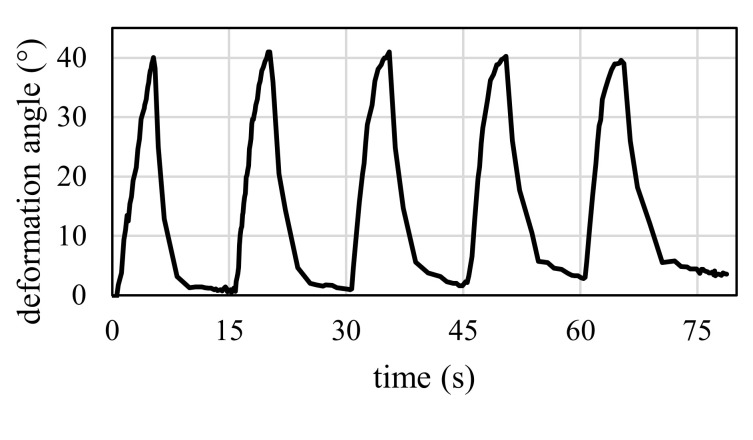
Deformation angle results of V1.1.

All specimen variants show the expected bending deformation behavior when activated (Figure 13). As in [6], a drift during deactivation is observable with all specimens, leading to decreasing setback angles. The effect can be explained by defects of the specimens and a setting effect of the SMA, which was observed in [6] as well. For V1.1 (Figure 13a), significant curling of the free edges is observed, impeding the overall achievable deformations. The SMA loop shape at the free end causes curling, which makes the SMA act locally with its contraction force in a 90° direction when activated. This effect may be of interest for future applications such as soft grippers. However, it is undesired for the goals of this work. Therefore, fixation screws are added to the SMA loops at the free specimen end, reducing the 90° pull effect and improving achievable deformation to a maximum angle of 41.0° (cf. Figure 13c). However, it is assumed that the deformation angles for specimens without edge curling would be larger. The resulting deformation angles of V1.1 are displayed in Figure 14.

**Figure 15:**
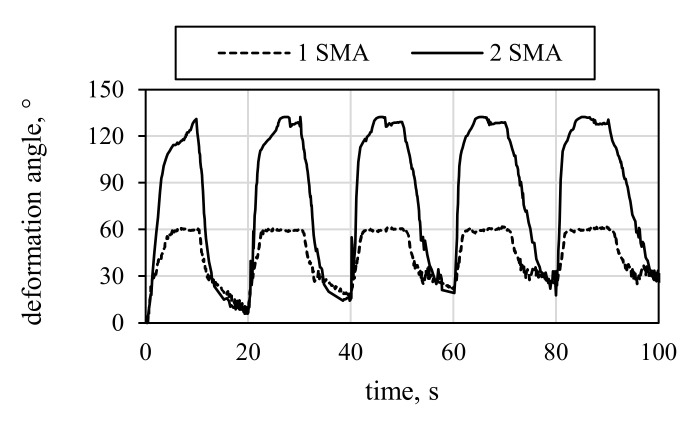
Deformation angle results of V1.2 for one SMA and two SMAs.

In contrast to V1.1, the variant V1.2 with higher stiffness shows significantly increased deformation angles (Figure 13b). The resulting deformation angles of V1.2 are shown in Figure 15. With one activated SMA (dotted line), the resulting deformation angle is greater than for V1.1, and even greater with two SMA (solid line), up to 132.3°. Two effects can explain the increase. Firstly, the additional textile layers prevent curling of the edges at the free end of the specimen, thus significantly reducing the loss of usable SMA stroke. Secondly, the additional textile layers increase the HSS bending stiffness, thus shifting the operating area of the SMA actuation force more prominently onto the LSS and thereby improving hinge functionality.

**Figure 16:**
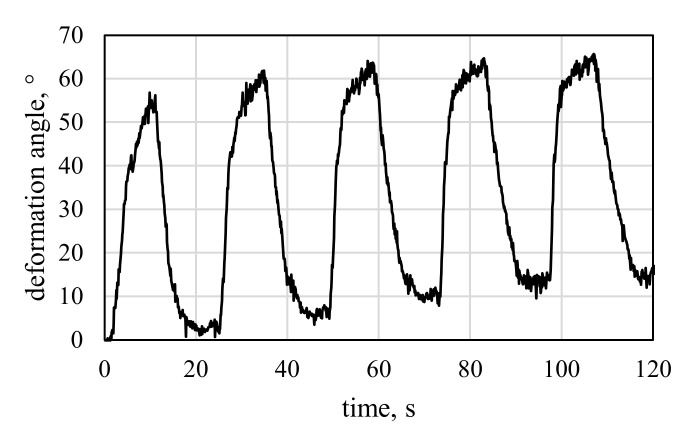
Deformation angle results of the activation test of V2, mode A—simultaneous activation of both hinges for 10 s.

**Figure 17:**
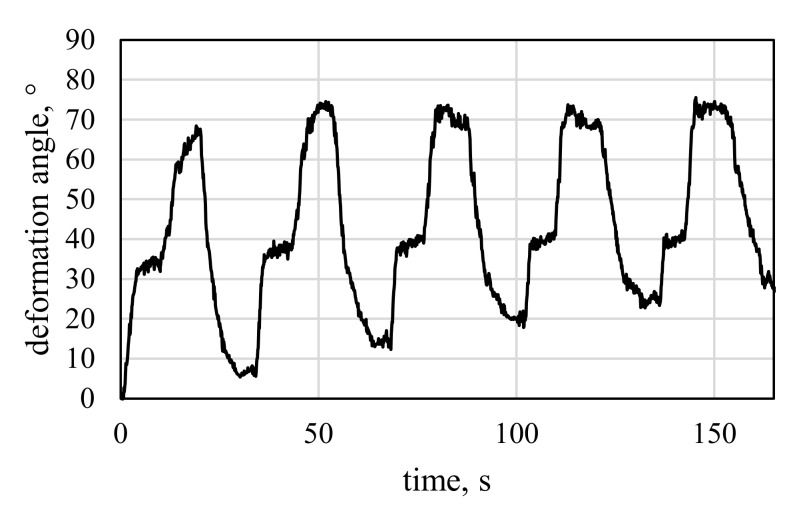
Deformation angle results of the activation test of V2, mode B—consecutive activation of the first SMA, followed by the second SMA after 10 s.

V2 in mode A shows simultaneous activation of both SMA sections in the specimen, while mode B displays the ability to trigger two sequential deformation states by consecutive activation of the two SMA sections. The results of the two different activation modes A and B (cf. Section 2.6) of V2 are displayed in Figure 16 and Figure 17, respectively.

Compared to mode B, the maximum angles achieved with mode A are about 10° lower. This effect can be explained by settling, which is typical for SMA-driven specimens, as was observed by e.g., Ashir et al. [11,12]. For V2, edge curling is barely observed compared to V1.1. One reason is the smaller diameter of SMA loops in the middle HSS, which makes the SMAs less susceptible to pull in a 90° direction. Another reason is the loop’s position in the middle of the specimen, where no free edges are present and continuous composite material surrounds the SMA loops.

The specimen V1.2 in turn shows higher deformation than V2, supporting the significance of uneven deformation distribution introduced in Section 2.2. It is assumed that a V2 specimen with additional stiffening of the HSS would show comparable or even larger deformations than V1.2.

Table 4 shows maximum deformation angles for all variants compared with those of unhinged specimens from a previous study [6]. The unhinged specimen has the same dimensions and fiber reinforcement as the HSS areas of specimens V1.1 and V2 (120 × 80 × 2.5 mm^3^, two layers of 410 tex GF fibers in the weft direction). The comparison of the maximum deformation angles reveals the difference between unhinged and hinged specimens.

### 3.4. Simulation Model Calibration

**Figure 18:**
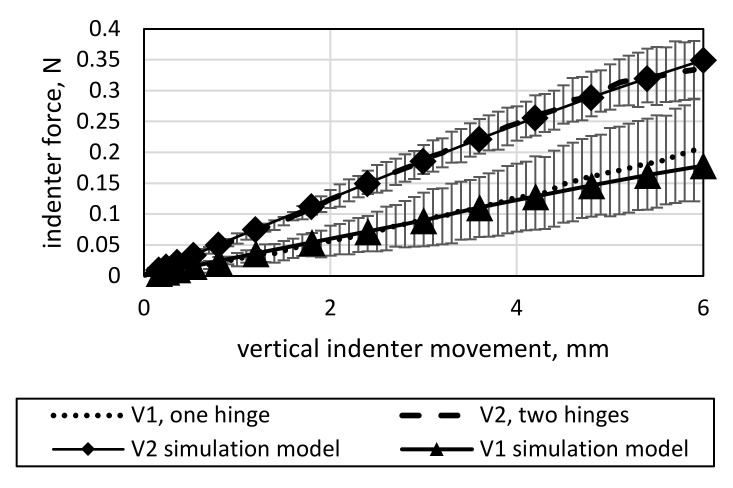
Four-point bending calibration results for the simulation models of V1 and V2.

The results of the model calibration for V1 and V2 to the four-point bending data of both variants are displayed in Figure 18.

It is assumed that material parameters determined for V1.1 and V2 are also suitable for V1.2. The material parameters of the orthotropic material model for the reinforcement fibers obtained from the calibration are displayed in Table 5.

An updated set of parameters was used for the Yeoh material model of the Sylgard rubber, providing a better fit for the experimental data than the dataset used in the previous work. The parameter set is displayed in Table 6.

### 3.5. Simulation Results

**Figure 19:**
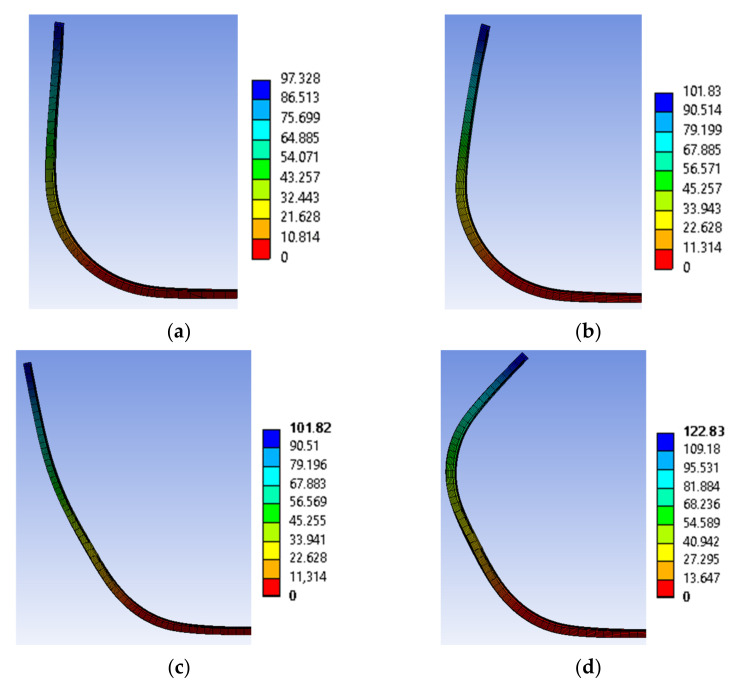
Simulation results of (**a**) V1.1, (**b**) V1.2, (**c**) V2 with one SMA activated and (**d**) V2 with both SMA activated. The color coding indicates the total deformation in mm.

The results of the simulations for V1.1, V1.2 and V2 are displayed in Figure 19.

The simulations of V1.1 and V2 show by factor ~2 higher deformations than the experiments. The high deviation can be explained by the edge curling present within the specimens, consuming a significant amount of usable SMA contraction. For V1.2, the simulation shows a lower deformation angle than the experiment by a factor of 0.8. It can thus be assumed that SMA transformation is not finished at the peak temperature of 110 °C used in the simulation. However, the model is unable to converge past 110 °C. A possible explanation for the large disparity of V1.2 is the modeling approach for IFRC, which may not be suited for large bending deformations above 90°. Calibration with experimental data is similarly challenging, since to our knowledge no experimental procedure exists for such large degree bending deformations. The maximum deformation angles of simulations and experiments are displayed in Table 7.

## 4. Conclusions

Flat knitting technology is well suited for producing biaxially reinforced knitted fabrics with integrated SMA wires and sectioned high and low stiffness areas, which can then be manufactured into hinged interactive fiber-rubber composites (IFRC) with active deformation capabilities. The solid-body hinges are able to increase deformation capability by concentrating contraction of the SMA wires on a smaller section of the specimen. The higher the stiffness difference between the hinge section and non-hinge section, the larger the achievable deformations. For fiber-reinforced rubbers, the difference in stiffness can be adjusted by varying the amount of fiber material. By choosing fibers with increased/reduced fineness, the fiber content can be adapted without the need for additional textile layers. The fineness of the chosen 410 tex GF material proved to be a good choice for creating high deformation specimens. In the warp direction, higher stiffness would be more feasible for reducing curling of the free edges and thus enabling larger deformations when activated. This can be achieved by using fiber material with higher fineness in the respective warp rows, again without the need to add more layers to the knitted fabric. Alternatively, edge curling can be reduced by decreasing the diameter of the turn-around curves of the SMA wire at the end of a weft row. This was shown in V1.2, where the stiffness gradient was increased by additional fibers, improving the maximum deformations.

Future work will apply hinged structures to practical applications such as grippers or locomotion scenarios, with the simulation models offering a practical tool for dimensioning and estimating structural behavior in different application scenarios. Since the hinge setup also induces local stress concentrations on both the fixation areas and the hinges, damages during cyclic actuation in these areas will be another subject of future research. Here, both damages to the IFRC and fatigue effects on the SMA are of interest.

## Figures and Tables

**Table 1:** Material properties.

Component	Property	Value
NiTi shape memory alloy wire *	Wire diameter (mm)	0.305
	Maximum actuation contraction	4.4 ± 0.2
	Forward transformation temperature range (°C)	78.1–97.7
	Pretreatment	Pre-strained
	Young’s modulus (GPa)	E_M_ = 32.5 E_A_ = 53.6
Silicone rubber	Elastic modulus (MPa)	1.1
	Fracture stress (MPa)	4.5
	Fracture strain (%)	86.8
	Density (g/cm^3^) **	1.05
Glass fiber yarn, 410 tex (GF-strong)	Elastic modulus (GPa)	80.2
	Yarn diameter (mm)	1.4
	Yarn count/fineness (tex)	410
	Density (g/cm^3^)	2.6
Glass fiber twisted twin-yarn, 2 × 136 tex *** (GF-faint)	Elastic modulus	67.2
	Yarn diameter (mm)	0.4
	Yarn count/fineness (tex)	2 × 136
	Density (g/cm^3^)	2.6

* Material provided by SAES^®^ Getter (20045 Lainate, Italy); ** Datasheet provided by ^®^DOW CORNING; *** Datasheet provided by CULIMETA.

**Table 2:** Layup for the composite forming process (VARI).

Layer	Description
1 Vacuum foil	Seals the layup vacuum-tight
2 Flow media	Supports the distribution of silicone and vacuum
3 Peel ply	Detaches release film and specimen
4 Reinforcement textile	Glass fiber knit cuttings
5 Mold	Sheet metal

**Table 3:** Composite specimen dimensions for V1 and V2.

Concept	Dimensions	Hinge Areas
V1	120× 80 × 2.5 mm^3^	45 mm
V2	120× 80 × 2.5 mm^3^	2 × 25 mm

**Table 4:** Maximum deformation angles of V1, V2 and a reference without hinges from previous study [6].

Specimen	Max. Deformation Angle [°]
V1.1, one hinge	41.0
V1.2, one hinge	132.3
V2, two hinges	75.5
Reference, no hinges	23.8

**Table 5:** Material parameters of the orthotropic material model used for the reinforcement fibers.

Parameter	Value
$E_{x}$ [GPa]	12.5
$E_{y}$ [GPa]	0.04
$E_{z}$ [GPa]	0.04
$\vartheta_{xy}$	0.22
$\vartheta_{yz}$	0.22
$\vartheta_{xz}$	0.22
$G_{xy}$ [GPa]	0.04
$G_{yz}$ [GPa]	0.02
$G_{xz}$ [GPa]	0.04

**Table 6:** Material parameters of the Yeoh material model used for the silicone rubber matrix.

Parameter	Value
C_10_ (Pa)	222,757.40564
C_10_ (Pa)	−42,093.2849079
C_10_ (Pa)	46,135.0702147
d_1_ (Pa^−1^)	0
d_2_ (Pa^−1^)	0
d_3_ (Pa^−1^)	0

**Table 7:** Deformation angles of all specimens in experiment and simulation.

Specimen		Maximum Deformation Angle, °
V1.1	Simulation	95.7
	Experiment	41.0
V1.2	Simulation	108.2
	Experiment	132.3
V2	Simulation	136.5
	Experiment	75.5

## Data Availability

The data presented in this study are available on request from the corresponding author.
